# *E. coli* Multiresistant Meningitis after Transrectal Prostate Biopsy

**DOI:** 10.1100/tsw.2006.362

**Published:** 2006-01-29

**Authors:** Diana Alecsandru, Israel Gestoso, Ana Romero, Alfonso Martinez, Ana García, Julio Lobo, M. Ruiz Yagüe

**Affiliations:** ^1^ Department of Clinical Immunology, Hospital Clínico San Carlos, Madrid, Spain; ^2^ Department of Microbiology, Hospital Clínico San Carlos, Madrid, Spain; ^3^ Department of Internal Medicine III, Hospital Clínico San Carlos, Madrid, Spain; ^4^ Department of Neurology, Hospital Clínico San Carlos, Madrid, Spain

**Keywords:** prostate biopsy, antibiotic resistance, prophylaxis

## Abstract

*Escherichia coli* meningitis is a frequent pathology in children younger than 3 years old, but is an uncommon disease in adults. *E. coli* infection is the main cause of intrahospital bacteremia as a consequence of the employment of different medical procedures. Our patient, male, 69 years old, presented with fever, progressive difficulty in breathing, and shivers 24 h after transrectal prostate biopsy, with an absence of any other symptoms. He received prophylactic treatment with ciprofloxacin and later empirical treatment with ampicillin and tobramicin. After that, the patient presented with fever, headache, behavioral changes, somnolence, disorientation, a fluctuating level of conscience, cutaneous widespread pallor, and acute urinary retention. On physical exploration, we observed generalized hypoventilation, Glasgow 10, stiffness of the neck, inconclusive Kernig; the remaining neurological exploration was normal. Systematic of blood: leukocytes = 8,510/mm^3^ (94.5% polymorphonuclear), platelet = 87,000/mm^3^, pH = 7.51, pCO_2_ = 28.8 mmHg, pO_2_ = 61 mmHg, O_2_ saturation = 93.8%, and remaining values were normal. Chest X- ray, cranial CT scan, urine cultures were normal. Blood culture: *E. coli.* CSF: glucose <0.4 g/l, total proteins = 3.05 g/l, PMN = 7 cells. Microscopic examination of the CSF: Gram-negative bacilli; CSF's culture: abundant *E. coli.* The case of acute meningitis by multiresistant *E. coli* after transrectal prostate biopsy presented demonstrates that antibiotic prevention with ciprofloxacin is not absolutely risk free. Besides the use of antibiotic prevention for multiresistant microorganisms, the urologist and other physicians involved in the procedure must not forget that the rate of major complications of transrectal prostate biopsy is 1%, especially when it is performed in patients who will not benefit from that biopsy.

## INTRODUCTION

Bacterial meningitis is an infection of the leptomeninges (arachnoids and piamater) by diverse bacteria with an inflammatory infiltration of polymorphonuclear leukocytes with great mortality without an immediate and appropriate antimicrobial treatment[1]. The incidence varies depending on the age of the individual, the development of the immunity for different microorganisms, and the geographical zone.

Meningitis by *E. coli* (with capsular antigen K1) and *Streptococcus agalactiae* predominates in children younger than 3 years old as a result of the mother's transmission of contamination from the childbirth channel. In the first 10 years, the microorganisms involved are *Neisseria meningitidis, Haemophilus influenzae*, and *S. pneumoniae*; in adults: *S. pneumoniae* and *N. meningitides*, and, in immunodeficient patients, *Listeria monocytogenes, Nocardia* spp., and *Enterobacteriae*.

*E. coli* is the most frequent cause of some of the most common bacterial infections, including urinary tract infections, bacteremia, and the traveler's bacterial diarrhea. As a consequence of the metastatic dissemination of the bacteremia or of the contiguous dissemination, *E. coli* has been revealed in a wide variety of diverse infections: septic arthritis, endophthalmitis, suppurated thyroiditis, intra-abdominal abscess, spontaneous bacterial peritonitis, hepatic abscess, encephalic abscess, endocarditis, osteomielitis, prostatitis, sinusitis, septic thrombophlebitis, and others[2].

Transrectal prostate biopsy is the most efficient diagnostic method for prostate carcinoma, as it is tolerated well in the majority of the cases with only few complications. Vasovagal syncope, rectorragia, and infectious complications may appear, the latter being the most serious, despite the widespread use of the antibiotic prophylaxis[3].

We present a case of *E. coli* multiresistant acute meningitis after a transrectal prostate biopsy.

## PATIENT AND METHODS

The patient, a smoker, male, 69 years old, with cervical degenerative osteoarthritis and hiatal hernia treated with ibuprofen and acid pump inhibitor therapy, came to the Service of Urgency of a tertiary care medical center in Madrid with fever (39.5°C), progressive difficulty in breathing, and shivers 24 h after a transrectal prostate biopsy indicated by PSA high values. He did not show any other symptoms. The patient had received prophylactic treatment with ciprofloxacin 500 mg/12 h until 2 days before the transrectal prostate biopsy. No urine culture was taken before the procedure.

On examination, a generalized hypoventilation was observed, increased prostate volume, of normal (not painful) consistency to the palpation; the remainder of the physical exploration was normal. Empirical treatment was initiated with ampicillin and tobramycin with clinical improvement in the first 24 h after admission.

After 72 h of treatment, the patient presented with fever, holocraneal migrainous headache, one vomiting episode, and behavioral changes with initial aggressive attitude followed by somnolence, disorientation, and a fluctuating level of conscience, cutaneous widespread pallor, intense perspiration, and acute urinary retention. On physical exploration, arterial pressure = 170/100 mmHg, T = 37.8ºC, cardiac frequency = 100 bpm, generalized hypoventilation, pain in the right hipochondrium, without signs of the venous thromboembolic disease, Glasgow 10, stiffness of the neck, inconclusive Kernig; the remainder of the neurological exploration was normal.

## RESULTS

The out-of-range results of the analytical parameters were: $[K^+]$ = 2.8 mEq/l, systematic of blood: leukocytes = 8,510/$mm^3$ (94.5% polymorphonuclear), platelet = 87,000/$mm^3$, pH = 7.51, $pCO_2$ = 28.8 mmHg, $pO_2$ = 61 mmHg, % Saturation $O_2$ = 93.8%, activated partial thromboplastin time = 39 seconds, INR = 1.3, prothrombin activity = 69.2%, and remaining values were within the normal range. ECG*:*

rhythmic 100 bpm, right bundle branch block. Chest X-ray, cranial CT scan, urine cultures were all normal. Blood culture: *E. coli* resistant to ampicillin, gentamycin, ciprofloxacin, and tobramycin, and sensitive to amikacin, cefotaxime, and piperaciclin-tazobactam.

The initial antibiotic therapy was changed to imipenem and a lumbar puncture was performed. Microscopic examination and culture of the CSF were performed: CSF: glucose <0.4 g/l, total proteins = 3.05 g/l, PMN = 7 cells. Microscopic examination of the CSF: Gram-negative bacilli. CSF's culture: abundant *E. coli* resistant to first-generation cephalosporin, gentamycin, amoxicillin-clavulanic, ciprofloxacin, tobramycin, and amoxicillin, and sensitive to cotrimoxazole, cefotaxime, and cefuroxime.

Cerebral magnetic resonance is performed: compatible with meningitis with images similar to vasculitis. Ventriculitis with plexitis.

Due to the bad clinical evolution, the patient was moved to the Critical Care Unit and the treatment was changed to cefotaxime. The final diagnosis was meningitis by multiresistant *E. coli* after transrectal prostate biopsy. The patient evolved towards disorientation and left-side hemiparesia. The result of the prostate biopsy was benign prostate hypertrophy.

## DISCUSSION

In this report, we added one case of meningitis by *E .coli* to the few previously described. Only one case with similar characteristics has been reported in the international literature[4] and another has been published recently in our country[3]. Meningitis by *E. coli* in developing countries[5] has been described in another context, in immunodeficient individuals[6] and after neurosurgery[7].

Generally, blood invasion by *E. coli* triggers sequential biological responses of the host against the pathogenic invader or its constituents (e.g., LPS) that determine the clinical progression from a syndrome of systemic inflammatory response (SSIR = fever or hypothermia, tachycardia, tachypnea, and leukocytosis) up to sepsis syndromes, severe sepsis, and septic shock[8].

However, the normal immune response can promote the disease when the inflammatory reaction is exaggerated in an attempt to stop the bacterial microorganism. Complement activation, cytokines liberation (e.g., TNF), leukocytes mobilization, platelet activation, and coagulation cascade are mediating factors of the lethal manifestations of the endotoxemia, including the extravasation of liquid and circulatory collapse, tubular renal necrosis, severe acute respiratory syndrome of the adult (when the microorganism is present in the CSF), and meningitis.

For meningitis to occur via blood infection, a previous bacteremia and hematoencephalic barrier destruction are required. The meninges are not usually affected by bacteremias, and probably elevated numbers of bacteria or a determined microorganism tropism is necessary to cause the disease. When microorganisms eventually invade the meninges and CSF, the initial growth is guaranteed because this space has few antibacterial defenses (phagocytes, complement, and immunoglobulin). Later on, the alteration of the hematoencephalic barrier allows inflammatory cells (neutrophils in this case), proteins, and other solutes to pass to the central nervous system.

Transrectal prostate biopsy is the most efficient diagnostic method for prostate carcinoma as it is tolerated well in the majority of the cases with few complications. The indications for an initial prostate biopsy are strongly influenced by digital rectal examinations, PSA (prostate-specific antigen) levels, and the PSA-related parameters of velocity, density, and percent free[9] (total PSA [PSAt] >10 ng/ml; PSAt equal to 4–10, 2.6–3.9, < or =2.5 ng/ml, and PSA F/T <25, <20, and <15%, respectively)[10].

Vasovagal syncope, rectal hemorrhage, and infectious complications may appear, with the latter being the most serious, despite the widespread use of the antibiotic prophylaxis as occurs in our case.

Although the indication of antibiotic prevention in urologic procedures (cystoscopy, etc.) in patients without bacteriuria is still a controversial issue, the justification for its use in the transrectal prostate biopsy is unanimously accepted and it is routinely performed with ciprofloxacin and a mechanical preparation of the rectum by enemas.

The case that we present in this report of acute meningitis by multiresistant *E. coli* after transrectal prostate biopsy demonstrates that the antibiotic prevention with ciprofloxacin is not absolutely risk free due to the frequent presence of multiresistant microorganisms in the hospital medium.

*E. coli* resistance to ampicillin, cotrimoxazole, ciprofloxacin, and gentamicin was found in 58, 33, 17, and 6.4% of isolates, respectively, in our area. Extended-spectrum beta-lactamase production was detected in 30 strains (1.5%). Ciprofloxacin resistance was higher in isolates from men and in-patients than in those from women and out-patients ($p < 0.001$). Multidrug resistance was present in 14% of *E. coli* isolates; the most prevalent phenotype was resistance to ampicillin, cotrimoxazole, and ciprofloxacin, which was detected in 59% of multiresistant strains and in 8% of strains overall[11].

In spite of the low morbi-mortality of prostate biopsy, it is necessary to keep the infectious risks in mind and the possibility of producing a bacteremia resistant to the antibiotic therapies (prophylactic and empirical) used until now, and the appearance of severe bacterial meningitis with a high mortality by the mechanisms previously described.

Besides the use of antibiotic prevention for multiresistant microorganisms, the urologist and other physicians must not forget that the rate of major complications of transrectal prostate biopsy is 1% (especially when it is performed in patients who will not benefit from that biopsy).

## ACKNOWLEDGMENT

To TheScientificWorldJOURNAL editor for handling our manuscript.
